# Neuromodulation in Intractable Epilepsy Through Responsive Vagal Nerve Stimulation: A Three-Year Retrospective Study at the University of Texas Medical Branch, Galveston

**DOI:** 10.7759/cureus.18698

**Published:** 2021-10-12

**Authors:** Joseph Villarreal, Vijaya Lakshmi Valaparla, Kyra Curtis, Neeharika Thottempudi, Sama Elrahi, Andrea Gil Guevara, Bhanu Gogia, Ruiqing Sun, Todd Masel, Prashant Rai

**Affiliations:** 1 Department of Neurology, University of Texas Medical Branch, Galveston, USA; 2 Department of Neurology, Beth Israel Deaconess Medical Center, Harvard Medical School, Boston, USA

**Keywords:** antiseizure medication, seizures, neuromodulation, epilepsy, vagus nerve stimulator

## Abstract

Background: Vagus nerve stimulation (VNS) functions through neuromodulatory mechanisms to provide quality of life improvements to those with drug-resistant epilepsy. Responsive VNS (rVNS) generators are designed to further reduce seizure burden by detecting ictal tachycardia and aborting seizures soon after their onset.

Methods: Electronic medical records were accessed from January 2015 to December 2018 to identify patients with epilepsy managed with rVNS generators. Data were collected on seizure burden before and after rVNS implantation. Seizure burden was compared using t-tests, and monthly seizure reductions were gauged with the McHugh scale. Twenty-seven individuals met inclusion criteria; 10 were eliminated due to prior VNS implantation or undocumented seizure frequencies.

Results: The average seizure burden prior to rVNS implantation was 24.78 seizures/month. Following generator placement, the mean seizure frequencies at three months, six months, 12 months, and 18 months were 6.81, 16.57, 5.65, and 5.78 seizures/month, respectively. However, despite documented reductions in the average monthly seizure frequency, we found no statistically significant differences in seizure frequency relative to baseline.

Conclusion: While many participants showed individual reductions in seizure burden, this study was unable to definitively conclude that rVNS therapy leads to statistically significant reduction in seizure burden.

## Introduction

Epilepsy has the potential to severely impair a patient’s quality of life; therefore, several pharmacological modalities have been synthesized to allow a reduction in seizure burden. However, despite being on multiple antiseizure medications (ASMs), more than one million people worldwide remain intractable to medical therapy [1].

A diverse array of nonpharmacological treatments exists for individuals with drug-resistant epilepsy, each best suited for a particular patient presentation. Conditions like mesial temporal sclerosis and lesional focal epilepsy identified on functional MRI (fMRI), EEG, or by depth electrode are good candidates for surgical management. However, it is curative only in up to 70% of these individuals [2,3]. Their use is also limited by the ability to locate a seizure focus and safely remove aberrant tissue. Neuromodulation is an important modality to consider in patients with intractable epilepsy to reduce their seizure burden [2,3].

After its initial approval for drug-resistant epilepsy in 1997, vagus nerve stimulation (VNS) has been found to be effective in the long term in providing good seizure control and potential quality of life improvements [4-6]. VNS technologies have also been well-tolerated and have yielded significant improvements in seizure burden, with a 51% average reduction in seizure frequency one year after device implantation [7-9].

VNS technologies continue to evolve since their introduction three decades ago. Literature has established an association between sudden increases in heart rate and seizure onset, making tachycardia a surrogate marker for seizure activity [1,10]. Newer responsive VNS (rVNS) generators, such as the AspireSR (Cyberonics, Houston, Texas, USA), take advantage of this seizure marker to abort breakthrough seizures [11-15]. Specifically, the rVNS incorporates a closed-loop feature, known as Automatic Stimulation Mode (AutoStim), to send electrical impulses to the left vagus nerve upon the detection of ictal tachycardia, allowing vagal stimulation shortly after seizure onset. This prompts a reduction in the severity and duration of seizures as compared to traditional VNS therapy.

Many studies have established the efficacy of older VNS technologies in reducing seizure burden. Although newer technologies that utilize the AutoStim mechanism sound more efficacious theoretically, evidence is still evolving. A few studies have reported improved seizure control after replacing traditional VNS with AutoStim [12,16]. Additionally, a handful of studies that evaluated patients with newly implanted AutoStim showed up to 50% reduction in seizure burden in up to 60% of the patients in long term [17]. 

This retrospective study aims to analyze the efficacy of newly implanted rVNS devices that use AutoStim technology through changes in seizure burden following generator implantation in a cohort of patients treated for intractable drug-resistant epilepsy at our institution.

## Materials and methods

Electronic medical records were accessed from January 2015 to December 2018 to identify patients with a history of epilepsy managed with the AspireSR pulse generator at our institution. Records of selected patients were retrospectively reviewed, and data were collected on VNS implantation date, seizure type, seizure burden prior to rVNS implantation, and seizure burden at multiple time points post-VNS implantation. Seizure burden before and after rVNS placement was then compared using t-tests, and monthly seizure reductions were gauged with the McHugh scale. P values <0.05 were considered statistically significant.

Initial review yielded 27 patients with intractable epilepsy managed with AspireSR pulse generators who had at least 18 months of follow-up. Of the 27 individuals who met inclusion criteria, eight were eliminated from the study due to unknown or confirmed prior traditional VNS exposure. Two additional individuals were eliminated due to undocumented seizure frequency before rVNS initiation. The remaining 17 patients were the focus of this study.

## Results

Patient demographics

Demographic variables were extracted from medical records of patients treated with rVNS therapy. Of the 17 patients analyzed, eight were male and nine were female. The mean age of individuals within the study cohort was 40.24 ± 0.78 years. Sixteen patients had a history of generalized seizures, while 10 had previously experienced focal seizures. All 17 patients had their epilepsy medically managed at study onset; six patients had ASM changes during the 18-month follow-up period. Table 1 summarizes the characteristics of patients treated with rVNS therapy within our institution.

**Table 1:** Patient demographics Except age, all values are expressed in frequency (n). ASM: antiseizure medication. ^a^Age expressed as mean ± standard error in years. ^b^Generalized involves tonic-clonic, atonic, and absence seizures. ^c^Focal involves focal awareness, focal impaired awareness, and awareness unknown.

Patient demographics	
Age^a^	40.24 ± 0.78
Sex	
Male	8
Female	9
Type of epilepsy	
Generalized^b^	16
Focal^c^	10
Managed with ASMs at study onset	17
ASM changes during follow-up period	6

Patient response to rVNS

In the study population, the average seizure burden prior to rVNS implantation was 24.78 seizures/month, with only one seizure-free individual (range: 0-210). Following rVNS generator placement, the mean seizure frequencies at three months, six months, 12 months, and 18 months were 6.81, 16.57, 5.65, and 5.78 seizures/month, respectively. However, despite documented reductions in the average monthly seizure frequency, as well as all patients reporting subjective improvements in their seizure burden, we found no statistically significant differences in seizure frequency at three months (P = 0.13), six months (P = 0.12), 12 months (P = 0.12), or 18 months (P = 0.06) relative to baseline. Table 2 displays average seizure frequencies at multiple time points following rVNS placement.

**Table 2:** Seizure frequencies following rVNS placement rVNS: responsive vagus nerve stimulation.

Time from rVNS placement	Mean seizure frequency ± SE (seizures/month)	P value
Before rVNS	24.78 ± 13.97	-
Three months after placement	6.81 ± 5.69	0.13
Six months after placement	16.57 ± 9.79	0.12
12 months after placement	5.65 ± 3.80	0.12
18 months after placement	5.78 ± 3.54	0.06

**Figure 1:**
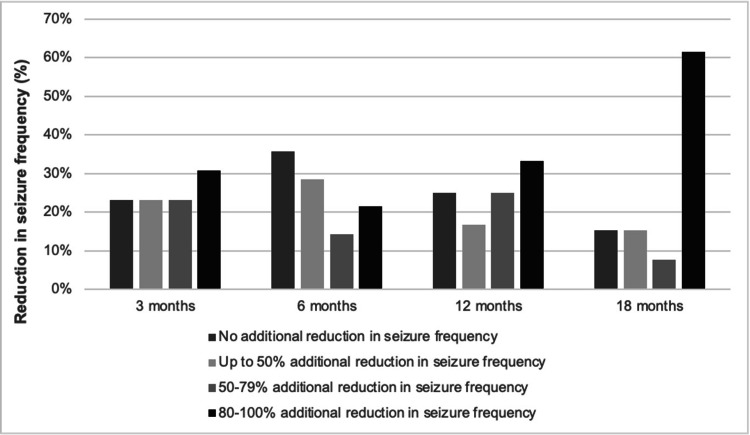
Additional reduction in seizure frequency following rVNS placement in VNS-naïve patients rVNS: responsive vagus nerve stimulation, VNS: vagus nerve stimulation.

The McHugh scale was used to classify monthly seizure reduction at three months, six months, 12 months, and 18 months post-rVNS generator placement. As magnet-stimulated VNS discharges were not central to this study, patients were solely divided into Class I (80%-100% additional reduction in seizure frequency), Class II (50%-79% additional reduction in seizure frequency), Class III (<50% additional reduction in seizure frequency), and Class V (no additional reduction in seizure frequency). We found that three months after initiating rVNS therapy, 30.7% of patients were Class I, 23.1% were Class II, 23.1% were Class III, and 23.1% were Class V. Similarly, at six months, 21.4% were Class I, 14.3% were Class II, 28.6% were Class III, and 35.7% were Class V. At 12 months, 33.3% were Class I, 25% were Class II, 16.7% were Class III, and 25% were Class V. Finally, at 18 months, 61.5% were Class I, 7.7% were Class II, 15.4% were Class III, and 15.4% were Class V. Figure 1 displays seizure frequency trends in accordance with the McHugh scale.

Safety outcomes after rVNS implantation

This study cohort’s average follow-up duration after rVNS implantation was 27.79 months. Of the 17 patients evaluated, only six reported adverse side effects during this time frame. Specifically, five patients reported a transient tickling sensation in their throat, while two endorsed episodic cough. No additional side effects were noted.

## Discussion

Since its inception, VNS therapy has held promise as an effective means of reducing seizure burden in patients with drug-resistant epilepsy who are poor surgical candidates. Incorporation of the closed-loop AutoStim feature expands on neuromodulatory mechanisms of older VNS technologies to more readily detect and abort breakthrough seizures [17]. The efficacy of rVNS generators is a subject of interest; thus, we sought to evaluate seizure burden following generator placement at our institution.

The efficacy of rVNS therapy

Analyzed patients were VNS naïve prior to implantation of rVNS generators. Additionally, all patients were medically managed with antiseizure medications; one patient was documented as seizure free prior to VNS therapy. This patient underwent generator placement due to ASM-related side effects. After 18 months of rVNS therapy, 69.2% (n = 9) of study participants had ≥50% reduction in seizure frequency, although no statistically significant changes in seizure frequency were detected. Tzadok et al. reported ≥50% reduction in seizure frequency in 62% of VNS-naïve patients at a mean follow-up of 13 months [15], results similar to those obtained in this study.

While many patients may experience side effects following VNS implantation, severe complications, such as infection, dysphagia, and vocal cord palsy, are rare, occurring in approximately 2% of patients [7,11]. The frequency of side effects in this study was consistent with rates from established literature, with 35.3% reporting either a tickling sensation in their throat or an episodic cough [11]. Additionally, no patients in this study experienced life-threatening adverse events. These findings suggest that rVNS therapy is a safe, and potentially efficacious, therapy for the management of epilepsy. Thus, further study of rVNS technologies is warranted.

Limitations

The current study had several limitations. First, six of the 17 individuals included in the study had changes to their antiseizure medication regimen within 18 months of rVNS placement. Alterations to ASMs may have introduced additional confounding variables, making it difficult to prove that changes in seizure burden were solely attributable to rVNS implantation. Additionally, missing information may have slightly skewed results of the study, decreasing the value of conclusions drawn. While all 17 study participants had their monthly seizure frequency recorded prior to rVNS therapy, three to five individuals were missing monthly seizure frequency data at each follow-up. As available data were collected during clinic visits, our conclusions may be further limited by recall bias. Finally, this single-institution retrospective review had a small sample size, which may have made it more difficult to fully evaluate the efficacy of newer rVNS technologies.

## Conclusions

This retrospective study aimed to evaluate the efficacy of responsive VNS devices in VNS-naïve patients. While many participants showed individual reductions in seizure burden, this study was unable to definitively conclude that rVNS therapy leads to statistically significant reduction in seizure burden, likely due to a small population size. Despite our study’s inability to detect significant changes in seizure burden following rVNS implantation, we envision rVNS technologies playing an essential role in the future management of epilepsy.
